# Reducing Heart Failure Readmissions Through an Advanced Practice Nurse–Led Clinic With Navigator Support

**DOI:** 10.1016/j.jaccas.2025.105663

**Published:** 2025-11-05

**Authors:** Tochi Okwueze, Amy Brownell

**Affiliations:** a Heart Failure Clinic, Ascension Illinois, Heart & Vascular, Elk Grove Village, Illinois, USA; b CV Service Line, Ascension Illinois, Heart & Vascular, Elk Grove Village, Illinois, USA

**Keywords:** acute heart failure, health care economics, lifestyle, secondary prevention

## Abstract

**Background:**

Acute heart failure is a leading cause of hospital readmissions, with nearly 20% rehospitalized within 30 days. Our institution's rate was over 27%, exceeding the national average.

**Project Rationale:**

To reduce readmissions, we launched an Advanced Practice Nurse-led clinic supported by Heart Failure Navigators. The strategy focused on lifestyle education, proactive monitoring, and coordinated care.

**Project Summary:**

Between 2021 and 2024, we followed up with adult patients discharged with New York Heart NYHA functional class II to IV heart failure, implementing telemonitoring, lifestyle coaching, guideline-directed medical therapy, and proactive outreach by Heart Failure Navigators.

**Take-Home Messages:**

Advanced practice nurses, with navigator support, reduce readmissions, boost medication adherence, and improve quality of life and outcomes.

## Graphical Abstract

**Figure:**
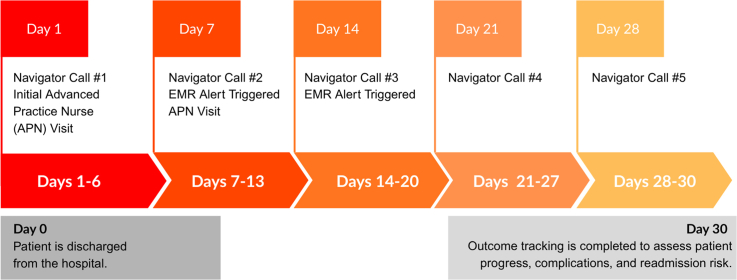

Heart failure is a chronic condition, but hospital readmissions are not inevitable. Almost 20% of patients return to the hospital within a month of discharge^1^, and the cost, financially and in quality of life, is high. At our hospital, the rate was even higher, at 27%. We knew we needed a new approach that helped patients stay on track once they left our care.

**Take-Home Messages**

• Navigator-supported care within an APN-led framework reduces readmissions, improves adherence, and enhances patient quality of life.
• This structured model exemplifies meaningful care transformation and holds promise for widespread adoption in heart failure management.

## Project Rationale

Life after HF hospitalization can be overwhelming. Patients often face gaps in follow-up, challenges managing medications, and uncertainty about when to seek help.^2^ We developed an outpatient model where advanced practice nurses (APNs) worked side-by-side with Heart Failure Navigators to guide patients through those first critical weeks, making sure they had the tools, knowledge, and support to manage their condition at home.

## Methods

This study was conducted as a retrospective analysis rather than through prospective patient enrollment. We reviewed all patients with a documented diagnosis of HF who were readmitted for HF-related complications between 2021 and 2024. A total of 90 patients were identified: 27 in 2021, 25 in 2022, 22 in 2023, and 16 in 2024. This cohort represents the captured population of HF readmissions at our institution during the study period. Nurse Navigators played a consistent role in care coordination and outcome monitoring throughout all 4 years.

• Patient Population and Characteristics

From January 2021 to March 2024, we reviewed data on adult patients who met the NYHA functional class II to IV HF criteria and were willing to participate in follow-up care at the outpatient heart failure clinic.

All patients included in the study had a documented diagnosis of HF and were readmitted due to HF-related complications. Although patients were not prospectively enrolled, the dataset captures the entire population of HF readmissions at our institution during the study period. Nurse Navigators were consistently engaged over the 4 years, providing care coordination and monitoring patient outcomes.

• Intervention design and care coordination

Telemonitoring: Daily weight checks, symptom reports, and virtual visits.

Education: Tailored guidance on diet, medication, and symptom management.

Medication review: APNs reconciled medications at the first visit and follow-ups.

Navigator outreach: Weekly phone calls to check symptoms, reinforce teaching, and coordinate appointments.

Electronic Medical Record (EMR) Communication: Real-time updates between navigators and providers for quick action when concerns arose.

• Clinical Metrics and Process Outcomes

30-day readmissions.

Medication adherence.

Patient engagement.

Quality-of-life scores (EQ-5D).

**Figure 1:**
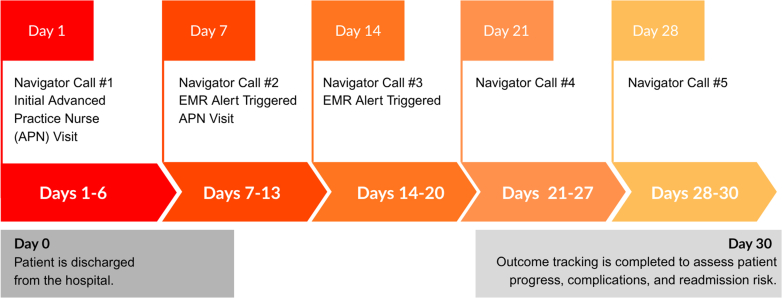
30-Day Post-Discharge Care Summary Day 0: Patient is discharged from the hospital. Days 1-30: Weekly navigator calls begin on Day 1 and continue every 7 days (total of 5 calls). Days 1-7: An APN conducts an initial follow-up visit within the first week. Day 14: An EMR alert is triggered to prompt provider follow-up, occurring 14 days after the APN visit, Third navigator call takes place. Day 30: Outcome tracking is completed to assess patient progress, complications, and readmission risk.

Demographics: average age 71 years; 56% male; 44% female; 38% Medicaid coverage (Figure 1).

## Results

The intervention yielded significant improvements across all metrics (Table 1). These results reflect the effectiveness of structured transitional care and align with national efforts to reduce HF readmissions.^3^^,^^4^

**Table 1:** Metrics

Outcome Measure	Initiation Phase (2021)	Wrap-Up Phase (2024)
30-Day readmissions	27 cases	16 cases
Readmission rate	>20% (2021)	5% (2024)
Medication adherence	Baseline	+21% improvement
EQ-5D quality-of-life score	Baseline	+8-point increase
Navigator engagement (avg. calls)	1.2/mo	3.4/mo

## Discussion

The APN-led clinic with navigator support demonstrated measurable success in reducing readmissions and improving patient outcomes. Several factors contributed to this.

## Continuity of Care

Navigators bridged gaps between discharge and outpatient follow-up.

## Patient Empowerment

Education and monitoring fostered self-efficacy.

## Timely Intervention

Electronic Medical Record alerts enabled rapid provider response to symptom escalation.

Two nurse navigators were already employed at the institution prior to the initiation of this project and provided care coordination and follow-up support for all patients included in the study. No additional staff were hired specifically for this research. For scalability, we estimate that programs managing 25 or more patients daily would warrant at least one full-time nurse navigator, with further staffing needs determined by patient complexity and available institutional resources.

APNs billed appropriate Evaluation and Management codes (eg, 99213-99215) as well as Chronic Care Management (99490, 99439, 99491) and Transitional Care management (99495, 99496) when applicable, in accordance with institutional and payer guidelines.^5^

This model is consistent with findings from Chow et al^2^ who emphasized the role of transitional care in improving HF outcomes. Moreover, our results support the CDC's recommendation for structured outpatient follow-up to reduce 30-day readmissions,^3^^,^^4^ also found that targeted interventions aligned with the Hospital Readmissions Reduction Program significantly improved outcomes in HF populations.

## Value Proposition

The program did not just work, it was cost-effective. By avoiding readmissions, we estimated an annual savings of about $340,000. The model is simple enough to scale and aligns with national goals for chronic disease management.^6^

## Limitations

While promising, the project had limitations:

Single-institution scope.
Potential selection bias (patients willing to engage in follow-up).
Limited generalizability to nonurban or resource-constrained settings.

Future studies should explore adaptation across diverse populations and settings.

## Conclusions

When patients leave the hospital with a solid plan, regular follow-up, and a team looking out for them, they are far less likely to end up back in the hospital. Our APN-led, navigator-supported program shows how structured transitional care can make heart failure management more effective and more.

## Funding Support and Author Disclosures

The authors have reported that they have no relationships relevant to the contents of this paper to disclose.
